# Neuralgic Operations

**Published:** 1853-08

**Authors:** 


### SELECTIONS.

From the American Journal of Med. Sciences.

*Neuralgic Operations.*

Dr. J. Mason Warren related the following case. The patient was a lady, 35 years of age, who, nine years before, was seized, while pregnant, with a severe pain in the tip of the forefinger of the right hand. The only cause she could attribute it to was the too free use of the needle. The pain had gradually increased, affecting the arm and shoulder, and, finally, other parts of the body on the same side. Every remedy, which the experience of distinguished surgeons in the vicinity could suggest, had been ineffectuelly tried. She was informed by Dr. W. that the success of an operation was doubtful. Aside from the above disease her health was otherwise good, and she was in good flesh. The appearance of the finger was somewhat red, and the motions impaired. It would not bear the slightest examination without the most excessive suffering, The patient being etherized, an incision was made a little in front of the inner aspect of the finger, the digital nerve exposed, and about half an inch of it excised. The same operation was repeated on the other side.

The operation had been followed by the most complete success; the patient was heard from some months afterwards perfectly free from a recurrence of her disease. Dr. W. referred to an operation similar to the above done by Dr. Buckminster Brown, and reported in the *Transactions* of the Society. These cases he thought interesting on account of the discredit into which the division of nerves for neuralgic affections had of late years fallen. At a subsequent meeting, he mentioned the case of neuralgia of the lower jaw, in which he had trephined that bone at its angle, and removed half an inch of the nerve, the operation being successful so far as he is acquainted with the after history.

Dr. Sargent, of Worcester, Associate Member of the Society, spoke of two cases in his own practice; the first he observed in a nervous female who had pricked the ulnar side of the palm of the hand near its articulation with the little finger; the neuralgic pain, in this instance, was evidently in the *skin;* very slight touch causing exquisite pain, while forcible, deep pressure did not; the touch of a feather upon the affected hand and arm produced acute suffering; the hand *springing up* convulsively at such touch; the pain first appeared in the seat of the injury. Dr. S. applied cantharides plaster, and then morphia to the cutis, repeating them three or four times; the treatment was followed with relief at each application, and after two or three weeks there was no recurrence of pain. The same patient afterwards injured the other hand with a pin, neuralgic pain of like kind supervened; similar treatment was followed speedily by a like result. Dr. S. alluded to analogous cases of long persistence, in which atrophy of the affected parts occurs; he has met with such; of neuralgia *confined to the skin* he had seen but two or three cases. A few years since, Dr. S. saw a man who had severe neuralgic pain supervene in the cicatrix of a cut which he had received on the dorsal aspect of one of the thumbs near the nail; a touch upon the scar caused great pain, which extended up the arm; blisters were ineffectual; an incision was productive of immediate and entire relief.

Dr. Parkman mentioned the case of a female of middle age, wounded by a needle in the ball of the thumb; spasmodic pain supervened extending to the entire arm, with severe spasms of the limb; free crucial incision afforded complete relief; the affection was of two or three years' continuance.

Dr. Brown remarked that, in the case reported by him and referred to by Dr. Warren, morphia applied to the blistered surface, and, indeed, every other known remedy was wholly ineffectual; the excision of a portion of the nerve was at once and entirely successful; no return of the disease.

Dr. Hayward, Sen., spoke of a case in which, a piece of a nerve being removed, no relief was experienced. Dr. H. cited a case in which Dr. Warren, Sen., removed an inch of the ulnar nerve for relief of neuralgic pain consequent upon a bruise of the arm and injury to the nerve, not the least relief of the pain followed; the patient, a young woman, finally submitted to amputation of the limb.

Dr. Parkman said that, in a case in which he divided the nerve at the mental foramen, last summer, with apparantly permanent relief, the neuralgia has returned, and now pervades the entire face.

